# Cohesin mutations: contributors to myeloid malignancies

**DOI:** 10.18632/oncotarget.21050

**Published:** 2017-09-19

**Authors:** Alison E. Meyer, Sridhar Rao, Joseph B. Fisher

**Affiliations:** Department of Natural Sciences, Concordia University Wisconsin, Mequon, WI, USA

**Keywords:** cohesin, AML, epigenetics

Acute myeloid leukemia (AML) is a hematopoietic malignancy hallmarked by complex cytogenetics and a poor prognosis. A number of driver mutations that underlie AML leukemogenesis have been identified, however somatic mutations are found in a variety of combinations, resulting in a high degree of genetic complexity. Such genetic variability results in difficulties finding an appropriate therapy that will work for all patients, and therefore more “personalized” approaches need to be tailored to a patient’s specific genetic profile. However, significant headway must first be made in understanding the contributions that individual mutations make to leukemia development.

In an effort to understand how one set of mutations contributes to AML development, our lab has been focusing on the cohesin complex. Mutations in members of the cohesin complex, comprised of the core subunits *SMC3*, *SMC1A*, *RAD21*, and *STAG1/2*, occur in roughly 10% of AML patients, but until recently have not been thoroughly investigated (reviewed in [1]). This commentary serves as a summary of the ongoing investigations into how cohesin mutations contribute to AML and discusses how our current knowledge may lead to therapeutic interventions for patients suffering from cohesin-mutated AML.

Mutations within cohesin genes were identified in patients with AML in 2013 [2], with follow up studies indicating that these mutations occur early during the leukemogenic process and cause clonal dominance concordant with leukemogenesis [3]. Consistent with the genetic complexity of AML, disrupted cohesin function alone is insufficient to cause disease. However, combining cohesin heterozygosity with the AML-associated FLT3-ITD mutation is sufficient to drive AML [4]. Until recently, the mechanism by which cohesin mutations contribute to disease progression was unstudied. Using diverse approaches, our lab and others have observed that disruption of cohesin function increases the self-renewal capacity of hematopoietic stem and progenitor cells (HSPCs) and disrupts normal hematopoiesis due to altered differentiation [1,4–8].

The mechanisms by which cohesin mutations influence HSPC biology appear to be multifaceted and independent of the canonical role of the cohesin complex in regulating sister chromatid cohesion during mitosis. Interestingly, disrupted cohesin function has been shown to alter genome-wide chromatin accessibility, resulting in increased accessibility at binding sites for GATA2, RUNX1, ERG [6], GATA1 [5], and STAT5 [4]. We have now shown that cohesin can contribute to gene repression as well by binding the Polycomb Repressive Complex 2 (PRC2), which mediates the repressive histone mark H3K27me3, and regulating its genomic targeting [8]. As a consequence of altered PRC2 binding, a genome-wide reduction in the repressive histone mark H3K27me3 occurs. We have subsequently shown reduced H3K27me3 levels at the *HOXA7/HOXA9* locus in cohesin-depleted cells [8]. Increases in HOXA7/9 levels are critical for AML development and disease progression and are frequently elevated in AML patients. Additionally, HOXA7/9 overexpression contributes to the enhanced HSPC renewal phenotype [8].

It has become increasingly clear that the mechanism by which cohesin mutations contribute to AML is independent of the canonical role of cohesin. Cohesin mutations instead influence AML development by disrupting the genomic architecture and preventing the recruitment of transcriptional or epigenetic regulators to proper genomic loci. In keeping with this idea, our laboratory has endeavored to identify small molecule therapeutic agents that target epigenetic regulators based upon our findings of altered PRC2 targeting following cohesin loss. Such epigenetic therapies may be capable of ameliorating the leukemogenic effects elicited by altered cohesin function. *HOXA9* is known to be regulated by the opposing actions of PRC2 and the DOT1L and COMPASS complexes, which deposit the repressive mark H3K27me3 and the activating marks H3K79me2 and H3K4me3, respectively [1]. Small molecule inhibitors that target the DOT1L complex are currently in clinical trials. We are now investigating if cohesin-depleted HSPCs exhibit increased sensitivity to DOT1L inhibitors. Given that cohesin mutations are thought to occur early, it is reasonable to speculate that treating AML patients harboring cohesin mutations with DOT1L inhibitors would target both leukemic blasts and leukemic stem cells. Testing the efficacy of small molecule inhibitors in cohesin-disrupted HPSCs has implications for personalized medicine, whereby patients harboring cohesin mutations may be more susceptible to such treatments.

Given the diverse functions of the cohesin complex, it is likely that other mechanisms of cohesin-mediated leukemogenesis exist and can be exploited for therapeutic purposes. Until further investigations are undertaken to identify these additional mechanisms, drugs targeting epigenetic regulators are the closest to the clinic. With the knowledge available we hope to identify and evaluate clinically relevant therapeutic strategies for treating AML.
